# Cloning and Characterization of Glutamate Receptors in Californian Sea Lions (*Zalophus californianus*)

**DOI:** 10.3390/md8051637

**Published:** 2010-05-06

**Authors:** Santokh Gill, Tracey Goldstein, Donna Situ, Tanja S. Zabka, Frances M. D. Gulland, Rudi W. Mueller

**Affiliations:** 1 Health Canada, Toxicology Research Division, Banting Bldg, Tunney’s Pasture, Ottawa, Ontario, K1A 0L2, Canada; 2 The Marine Mammal Center, 1065 Fort Cronkhite, Sausalito, CA 94965, USA

**Keywords:** harmful algal blooms, domoic acid, Californian sea lion, conducting system, heart, immunohistochemistry, glutamate receptors

## Abstract

Domoic acid produced by marine algae has been shown to cause acute and chronic neurologic sequelae in Californian sea lions following acute or low-dose exposure. Histological findings in affected animals included a degenerative cardiomyopathy that was hypothesized to be caused by over-excitation of the glutamate receptors (GluRs) speculated to be present in the sea lion heart. Thus tissues from five sea lions without lesions associated with domoic acid toxicity and one animal with domoic acid-induced chronic neurologic sequelae and degenerative cardiomyopathy were examined for the presence of GluRs. Immunohistochemistry localized mGluR 2/3, mGluR 5, GluR 2/3 and NMDAR 1 in structures of the conducting system and blood vessels. NMDAR 1 and GluR 2/3 were the most widespread as immunoreactivity was observed within sea lion conducting system structures. PCR analysis, cloning and subsequent sequencing of the seal lion GluRs showed only 80% homology to those from rats, but more than 95% homologous to those from dogs. The cellular distribution and expression of subtypes of GluRs in the sea lion hearts suggests that exposure to domoic acid may induce cardiac damage and functional disturbances.

## 1. Introduction

Domoic acid is a potent neurotoxic analogue of the naturally occurring excitatory amino acid l-glutamate. This toxin enters the marine food chain following production by several genera of marine algae having worldwide distribution. Domoic acid was responsible for an outbreak of amnesic shellfish poisoning (ASP) in 1987 in humans [1–6]. The syndrome was named ASP due to the confusion, loss of memory and disorientation that developed in affected people [2–5]. Subsequently, it has been shown to affect numerous organisms and causes mortality in a variety of species, including sea birds, marine mammals and fish [7,8–16]. The common neurological signs of domoic acid poisoning, including pruritus, ataxia, tremors and seizures, have been documented in mice, rats, cynomologus monkeys and Californian sea lions (*Zalophus californianus*) [1–3,5,6,13,14]. In addition to the neurologic effects, experimental exposure in laboratory animals and natural exposure in humans resulted in gastro-intestinal disturbances, cardiovascular arrhythmias and collapse [3,17–19].

In 1998 and 2000, hundreds of free-ranging Californian sea lions, predominantly adult females, suffered from domoic acid toxicity during three harmful algal bloom events in central and northern California coastal waters. Heart pathology was reported in these acutely affected sea lions [8,11–13,15,16], and since then, a degenerative cardiomyopathy has been characterized in both males and females of all ages suffering from acute and chronic effects [16]. Gross pathology was non-specific and not present consistently. Histopathology was characterized by cardiomyocyte vacuolar degeneration and loss with adipocyte replacement that started at the base of the interventricular septum distal to the atrioventricular node and progressed distally and circumferentially, especially in the mid-myocardium to subendocardium. The features of the degenerative cardiomyopathy were not consistent with other known causes of heart lesions in sea lions and were less consistent with a centrally-mediated (brain-heart) mechanism. Thus, the cardiomyopathy was hypothesized to be caused by exposure to domoic acid activating the glutamate receptors that maybe present in the heart [16].

There are two groups of glutamate receptors identified: (1) ionotropic glutamate receptors (iGluRs) that are fast-acting and (2) metabotropic glutamate receptors (mGluRs) that mediate more long-term cellular function. Ionotropic receptors contain integral cation-specific ion channels and are subdivided according to their selective agonists into (1) *N*-methyl-d-aspartate (NMDA), (2) α-amino-3-hydroxy-5-methyl-4-isoxazole-propionic acid (AMPA), and (3) kainate (Ka) receptors. Activation of these receptors leads to the opening of a group of ion channels that are typified by their different permeabilities to Na^+^, K^+^, and Ca^2+^ [20–22]. The mGluRs are coupled to G-proteins and are subdivided according to agonist interactions and second messenger activation into (1) mGluR 1 and 5; (2) mGluR 2 and 3; and (3) mGluR 4, 6, 7 and 8. Activation of these receptors modulate the production of second messengers such as inositol phosphates and/or adenylate cyclase [3,21–23]. The potential toxic effects of glutamate and its analogues are not limited to the brain, as a recent review of the anatomical distribution of glutamate receptors demonstrated their presence in peripheral tissues, including the heart [3,17,18,24]. Also, there is accumulating evidence that GluRs mediate excitatory neurotransmission in peripheral neural and non-neural tissues and are involved in various organ/tissue functions and pathologies [17–19].

The objectives of this paper were to: (a) demonstrate the cell specific presence of the different subtypes of the glutamate receptors (GluRs) in sea lion heart using immunochemical methods; (b) to clone and sequence these GluRs; and (c) to ascertain whether the GluRs were involved in the cardiomyopathy using expression profiles for different subtypes of GluRs.

## 2. Results

**Figure 1:**
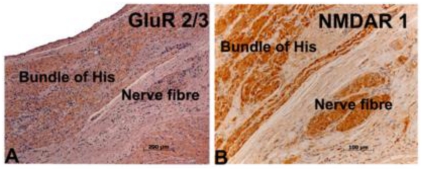
Photomicrographs of the heart tissue from animal No. 1 stained with polyclonal antibodies for glutamate receptors in microwaved paraffin sections (A). GluR 2/3 (0.008 μg/mL) and (B). NMDA R1 (0.01 μg/mL) both staining the bundle of His and nerve fibers.

Immunohistochemistry was performed on formalin-fixed archived heart tissues obtained from two control animals. Antibodies anti-GluR 2/3, anti-NMDAR 1, anti-mGluR 2/3 and anti-mGluR 5 were used and all four were immunoreactive with the bundle of His and the nerve fibres (Figure 1A to Figure 1B). The AV node and ganglia cells were immunoreactive for anti-NMDAR 1 and anti-GluR 2/3 antibodies. Cardiac blood vessels were immunoreactive for the four antibodies. Anti-Ka-2 antibody did not provide specific immunoreactivity at any dilution.

Molecular analysis was performed on RNA extracted from frozen heart tissues from three different control animals and from one heart obtained from a sea lion suffering from chronic effects from previous domoic acid toxicity showing both brain and heart pathology. Using primer pairs designed from several regions of the rat glutamate receptor subtypes, PCR conditions were optimized for each receptor using the RNA obtained from the rat heart. The correct amplified products were obtained. However, using these same conditions and the same primers, no PCR products were obtained using either total RNA or mRNA obtained from the sea lion hearts.

**Figure 2:**
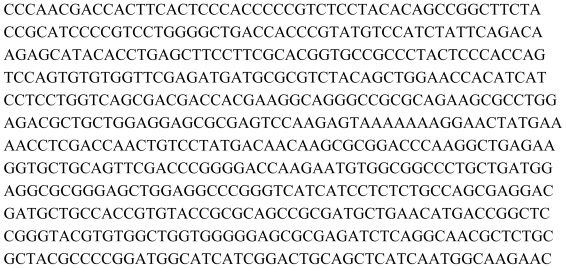

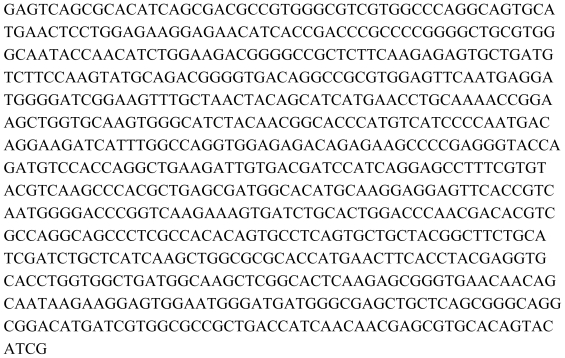
Sequence of the 1393bp fragment of the NMDAR 1 receptor from Californian sea lions.

**Figure 3:**
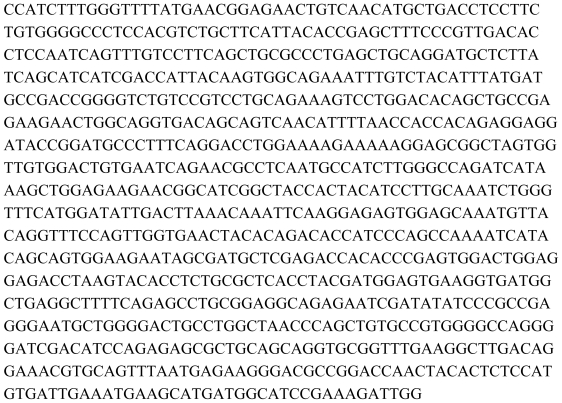
Sequence of the 927 bp fragment of the GluR 1 receptor from Californian sea lions.

**Figure 4:**
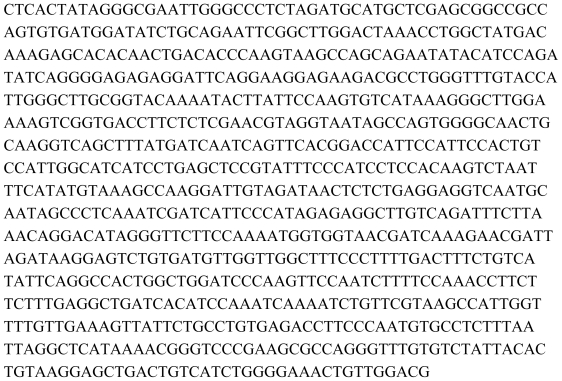
Sequence of the 840bp fragment of the GluR 6 receptor from Californian sea lions.

Hence, an alternate strategy had to be devised to clone the GluRs from the heart of sea lions for expression analysis. Three different primer sets (Table 1) were designed and synthesized from various conserved domains by aligning (using ClustalW2) the cDNA sequences of the dog, human, rat and mouse available from GenBank. Cloning of these receptors was only done from the rat, human and the seal lion. Using the PCR primers, the correct PCR fragments were obtained for all these three species. These fragments were all extracted from the agarose gel and cloned into the PCR2.1-TOPO TA-cloning vector. The clones corresponding to Ka-2, NMDAR 1, GluR 1, GluR 6 and mGluR 5 from all three species were sequenced. All sequences obtained from the clones corresponding to the rat and human samples were 100% homologous to their counterparts. The clones obtained from the sea lion hearts showed varying amounts of homology to the GluRs of the dog and the rat. NMDAR-1 (Figure 2) was 94% homologous to the dog (*Canis lupus familiaris lupus*; NM_001008717.1) and only 88% to the rat (*Rattus norvegicus*; NM_017010.1). GluR 1 (Figure 3) was 97% homologous to the dog (*Canis lupus familiaris lupus*; XM_546286.2) and 88% to the rat (*Rattus norvegicus*; NM_031608.1). The GluR 6 (Figure 4) was 97% homologous to the dog (Canus *lupus familiaris lupus*; XM_539059.2) and 92% to the rat (*Rattus norvegicus*; NM_019309.2).

Sequences corresponding to mGluR 5 were not obtained from sea lion tissues; however, those obtained from both rat and human RNA aligned with their respective sequences. For the Ka-2 receptor, several different size clones were obtained. All clones and the PCR fragment, which was extracted from the agarose gel, were sequenced. The clones showed varying homology to the Ka-2 sequence in GenBank. However, when the PCR product was extracted from the agarose gel and sequenced, the sequence was 100% homologous to the Ka-2 receptor of the mouse (*Mus musculus*; NM_008168.2) and the rat (*Rattus norvegicus*; NM_031508.2). This suggests that during the cloning process, the PCR fragment underwent rearrangement in the bacterial host [25].

## 3. Expression Analysis

**Figure 6:**
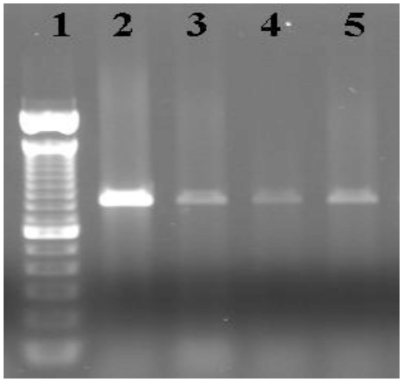
Agarose gel (1%) image showing results from the PCR fragment of the glutamate receptor subtype GluR 6. Lanes 2, 3, and 4 correspond to the PCR fragments obtained from control animals, and lane 5 corresponds to one domoic acid affected Californian sea lion. Lane 1 corresponds to the molecular weight marker. The size of the PCR product is approximately 840 base pairs.

PCR fragments for GluR 1 and GluR 6 were examined to compare levels of expression between the three control animals and one domoic acid-affected animal. Although both receptors were expressed in the left and right ventricles of the heart from each animal, there was varying amounts of expression among the control animals for both subtypes. Figure 6 shows the level of expression of GluR 6 for three different control animals (lanes 2–4) and one domoic acid-affected animal (lane 5). There was varying amounts of expression (as observed by the intensity of the PCR fragments in the agarose gel) among the control animals. This illustrates the inter-individual variation among the control animals and domoic acid-affected animal.

## 4. Discussion

In the CNS, GluRs have been shown to be the mediators for excitotoxic effects of excitatory amino acids (EAAs) and/or their structural analogues such as domoic acid. Although these receptors were once thought to be predominantly located in the CNS, recent evidence shows that they are also present in peripheral neural and non-neural tissues [17–19,24]. These GluRs in peripheral tissues are potential targets for the toxic effects of EAAs present in foods and the environment [17–19,24]. The association of arrhythmias and other cardiovascular symptoms in humans intoxicated with DA and on individuals susceptible to MSG prompted us to focus our attention to the investigation of GluRs in heart [1,3,17–19,24]. We have hypothesized that GluRs play a role in mediating the cardiac effects of these glutamate analogues. Hence, the observation of degenerative cardiomyopathy in sea lions intoxicated with DA further supports the view that DA may be cardiotoxic [3,4,16,18,19].

In order to determine whether the glutamate receptors were directly involved in the pathogenesis of the degenerative cardiomyopathy, the GluRs were localized to specific cellular structures using immunochemical methods. Antibodies to GluR 2/3, NMDAR 1, mGluR 2/3 and mGluR 5 were all immunoreactive with the bundle of His, the nerve fibres and the cardiac blood vessels. NMDAR 1 and GluR 2/3 were found in the AV node and ganglia in the hearts obtained from both normal sea lions and an animal showing degenerative cardiomyopathy. This specific cellular localization of GluRs to the different components of the conducting system supports that these receptors may play a role in cardiac physiology, and in particular may be involved in pathogenesis of the degenerative cardiomyopathy as a direct result of the over-excitation of GluRs in heart tissue. This is supported by the fact that the distribution of the cardiomyopathy was distal to the AV node and in close proximity to the major conducting system structures and often small to medium-sized blood vessels. From our immunohistochemistry data, these are the same regions in which the GluRs were localized. Domoic acid is known to exert its neurotoxic effects by binding primarily the NMDA, GluRs in the central nervous system causing increased intracellular calcium and eventually neuronal death through apoptosis [17,23,26]. Recent *in vitro* studies provide evidence suggesting that stimulation of NMDA GluRs in the cardiomyocytes may lead to apoptosis via a Ca^2+^, ROS, and caspase-3 mediated pathway [24,26]. This mechanism is consistent with the morphology of the cardiomyopathy, which had features of apoptosis, and cardiomyocytes immunoreactive to caspase-3 antibody [16]. Thus, these findings in conjunction with the clinical manifestation of unstable blood pressure and arrhythmias in humans with ASP and laboratory animals experimentally exposed to domoic acid [3–6,17,27] strongly suggest a functional role for these receptors in the heart. Zabka *et al.* [16] showed that severity of domoic acid-induced brain lesions and death did not always correlate in some sea lions, and thus suggested that the cardiomyopathy may be associated with functional cardiac disturbances that could be fatal.

In addition to the immunochemical localization of the glutamate receptors we performed expression profiles of two subtypes of glutamate receptors, GluR 1 and GluR 6 in three control sea lions and one domoic acid affected animal. As there was inter-individual variation in expression for both GluRs in the control animals and the domoic acid affected animal, it could not be ascertained if the GluRs were involved in cardiomyopathy. Hence, a larger number of tissues from both control and domoic acid-affected animals must be analyzed in order to correlate changes in expression with the distribution of the receptor subtypes.

## 5. Experimental Section

### 5.1. Materials and Methods

Tissues and sections were obtained from Californian sea lions that died while undergoing rehabilitation at The Marine Mammal Center, Sausalito, CA, USA. The control animals were euthanized while undergoing rehabilitation as a result of trauma, blindness or severe eye disease. The designation of “control” was based on the lack of domoic acid-associated ante- or post-mortem findings, as the history of domoic acid exposure in free living sea lions is unknown. The domoic acid affected animal was a juvenile male that restranded within a month of initial release following diagnosis and treatment for long term effects from previous exposure to domoic acid. This animal was euthanized shortly following readmission due to persistent neurologic disease. Gross and histological examination showed hippocampal atrophy consistent with exposure to domoic acid and also demonstrated a mild, acute degenerative cardiomyopathy.

### 5.2. Tissue Preparation and Immunohistochemistry

Immunohistochemistry for various subtypes of GluRs was performed on formalin-fixed archived samples of hearts to demonstrate their presence and distribution. The regions of heart examined were the mid-ventricle with distal bundle branches and peripheral nerves, subvalvular to the right atrioventricular (AV) valve with proximal bundle branches and peripheral nerves, and right AV valve with AV node and the Bundle of His. Tissues were embedded in paraffin and sections (4–5 μm) were mounted on silinated slides. The sections were deparaffinized and passed through a series of 100% ethanol. Slides were placed in 10 mM sodium citrate buffer (pH 6.0) and microwaved for antigen retrieval for two 3-minute periods at 450 W with gentle agitation (Kenmore 900 W or H2200/Energy Beam Science Inc.). Microwave-treated sections were washed in phosphate buffered saline (PBS) and blocked for endogenous avidin and biotin in a 0.5% hydrogen peroxide/100% ethanol solution. Slides were rinsed in 95% ethanol and then in running distilled water [18,19,24]. Immunohistochemistry was performed using the avidin-biotin method and the following primary polyclonal antibodies: NMDAR-1 (0.01 ug/mL; Chemicon International Inc., Temecula, CA, USA), anti-mGluR 5 (0.05 ug/mL; Chemicon International Inc.), anti-GluR 2/3 and anti-mGluR 2/3 (0.008 and 0.01 ug/mL, respectively; Chemicon International Inc.), and anti-Ka-2 (Upstate Biotechnology Inc., New York, USA). To obtain the optimal dilutions, a series of primary antibody dilutions, which ranged from 0.0005 to 0.02 μg/μL and were made in 15% normal swine serum [18,19,24]. After washing in PBS, slides were then incubated at room temperature in biotinylated F(ab’) swine-anti-rabbit secondary antibody (Dako Canada, Inc., Mississauga, ON, Canada) diluted in 15% normal serum. The slides were washed again in PBS, followed by an incubation of 30 min at room temperature in streptavidin (1:200) complex (Dako Canada, Inc.). Slides were washed again in PBS and 50 mM Tris buffer and then treated with 3′,3-diaminobenzidinetetrachloride (DAB 80 mg/72 uL of 30% hydrogen peroxide) in 400 mL 50 mM Tris buffer (pH 7.2). Finally, slides were rinsed in running tap water, counterstained with hematoxylin, rehydrated and coverslipped with Micromount medium (Surgipath Canada, Winnipeg, MB, Canada). Sections were stained with Mayer’s haematoxylin and eosin Y (H&E, Armed Forces Institute of Pathology, Washington, USA) for anatomical reference. Photographs were taken with an Axiophot Zeiss microscope (Germany) equipped with a digital camera.

The specificity of the antibodies and their binding capacity in formaldehyde fixed tissues were determined using peptide absorption [18,19,24]. Negative controls were evaluated with the diluent solution (Dako Canada, Inc.) containing 15% normal swine serum substituted for the primary antibody. Pre-absorption controls were prepared for all tissues tested. Peptides for mGluR 2/3, GluR 2/3 and NMDAR 1 were custom synthesized by Quality Controlled Biochemical, Inc. (Hopkinton, MA) using the peptide sequences provided by Chemicon International Inc. Each peptide ranging from 1.0 mg/mL to 0.004 mg/mL was incubated with the optimal concentration of the specific antibody at room temperature for 1 hour prior to placing the mixture on the slides. Rat brain slides at the level of the hippocampus were used as positive controls for the GluR antibodies.

### 5.3. Primer Design

Initial attempts to clone the glutamate receptors using primers based on the rat sequences were unsuccessful. This was speculated to be due to the: (1) low prevalence of GluRs, which then precludes cloning using total RNA and requires mRNA; and/or (2) the sequences of the GluRs from sea lions are sufficiently dissimilar to those from rat. Therefore, a search for evolutionary relatedness was performed. Based on mitochondrial gene sequences, Reyes *et al*. [28] concluded that Steller sea lions (*Eumetopias jubatus*) and dogs are related. Since both Californian and Steller sea lions belong to the family Otariidae, GluR sequences for several subtypes from the dog were included in alignments along with the cDNA sequences of the human, rat and mouse available from GenBank. The primers were designed using aligning (ClustalW2) dog, human, rat and mouse cDNA sequences. Three different primer sets (Table 1) were designed from various conserved domains. Sequences for Ka-2, GluR 2 and mGluR 5 were obtained and compared from human, dog and rat and were assigned the following accession numbers: Ka-2-human (accession number-NM_002088); dog (accession number-XM_541594); rat (accession number NM_017262); GluR 2-human (accession number-NM_000827); rat (accession number NM_031608); Dog (accession number -XM_546286); mGluR 5-Human (accession number-NM_000842); rat (accession number-NM_017012), and dog (accession number-XP_856685). Due to inter-species sequence variability for NMDAR 1, primers based on rat sequences (accession number NM_017010) were used to amplify a product from sea-lion mRNA.

### 5.4. PCR and Cloning

The RT-PCR, PCR, and cloning methods have been previously described [24]. In brief, total RNA was extracted from the frozen left and right ventricles using the Trizol reagent (Invitrogen, Burlington, ON, Canada) according to the manufacturer’s instructions. RNA was dissolved in DEPC water and examined by spectrophotometric quantification (Nanodrop ND-1000 Spectrophotometer, Thermo Scientific).

First strand cDNA synthesis was performed by using the Quantitect Reverse Transcription Kit (Qiagen; Mississauga, ON, Canada). Several attempts were made to amplify gene fragments of the different subtypes of glutamate receptors including NMDAR 1, Ka-2, GluR 1, mGluR 5 and mGluR 2 using three sets of primers (Table 1) for each subtype of GluRs. Conditions for amplifying glutamate receptors were optimized for the total RNA from the rat brain using a thermo-gradient (DNA Engine Peltier Thermal Cycler PTC-200, Bio-Rad Laboratories) from 52 °C–62 °C. PCR fragments were obtained from the cDNA made from the total RNA from rat tissue but no PCR fragments were visualized using total RNA extracted from sea lion hearts. Thus mRNA was extracted using the Oligotex kit (Qiagen Inc.) and RT-PCR and PCR were repeated using 100 ng of mRNA for the first strand synthesis. Using mRNA, PCR products successfully obtained and the appropriate size fragments were extracted using Qiagen gel extraction kit, and ligated into the PCR2.1-TOPO TA-cloning vector (Invitrogen Canada Inc.). The ligation mixture was transformed into competent cells of INVαF cells according to the manufacturer’s instructions (Invitrogen Canada Inc.). Recombinant clones were extracted and these were analysed using double restriction digest with Not 1 and BamH 1 flanking the PCR fragments.

### 5.5. Sequencing of Clones

Purified plasmid DNA (200–500 ng) was used as a template for cycle sequencing for animals Nos. 3–6 (Big Dye Terminator v3.1, Applied Biosystems, Mississauga, ON, Canada) using the M13 forward and reverse primers. Post-reaction clean-up was performed using a 96 well filter binding system (Montage SEQ96, Millipore, Etobicoke, ON) and fragments were resolved on an ABI 3130XL genetic analyzer running DNA Sequencing Analysis Software v5.2 (Applied Biosystems, Foster City, CA, USA). Individual sequencing runs were checked for quality using Sequencher v4.8 software (Gene Codes Corp. Ann Arbor, MI). Each plasmid was sequenced in both directions. The sequences were analysed using the Basic Local Alignment Search Tool (BLASTN) from the National Center for Biotechnology Information (NCBI) centre.

## 6. Conclusions

In this study we have shown using both immunohistochemistry and molecular biology, a number of iGluRs and mGluRs including-mGluR 2/3, mGluR 5, GluR 2/3, NMDAR 1, Ka-2, GluR 1 and GluR 6 were all expressed in the sea lion hearts. Since these receptors were present in the heart it was hypothesized that the domoic acid maybe directly responsible for causing cardiomyopathy which has been observed in earlier studies. Although, the immunochemical localizations supports this hypothesis, the expression profiles, due to the inter-individual variation, does not conclusively support this hypothesis. Hence, further experiments with domoic acid treated animals need to be performed in order to confirm the hypothesis that degenerative cardiomyopathy could be due to the direct interaction of domoic acid and the presence of glutamate receptors in the heart [16].

## Figures and Tables

**Figure 5:**
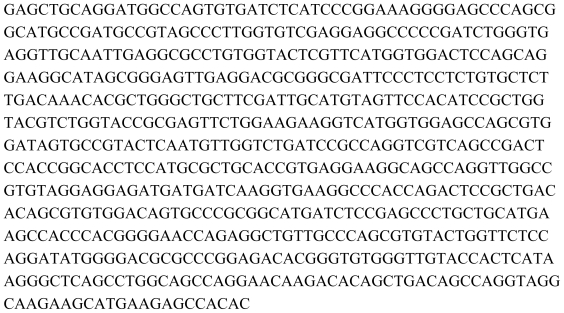
Sequence of the 875 bp fragment of the kainite receptor Ka-2 from Californian sea lions.

**Table 1:** Primers designed for cloning the subtypes of Glutamate Receptors from the Californian Sea Lion (*Zalophus californianus*).

Gene of interest	Forward Primer	Reverse Primer	Expected fragment size
GluR 6	GGACTAAACCTGGCTATGAC	CATGCAGCAAGGTTCTGAGC	840 bp
Kainate Ka 2	GCACATGGGCCGCAAGC	CAGCAATGATGAGGCCACAG	875 bp
GluR 1	GTTCCCAGTTCTCCAAAGGAG	R1-CAT TCC AGT AAC CGA TCT TTC GG R2-CAT TCC AGT AAC CAA TCT TTC GG	927 bp
mGluR 5	CTTCAGGCGAAGCATGAAG	GGTGGCGGCAGCGGATG	947 bp
NMDAR 1	CTAGCCAGGTCTACGCTATC	CTGGGAATCTCCTTCTTGACC	1393 bp

Note: For GluR 1, equimolar of the Reverse primers R1 and R2 were used.
